# Divergent Effects of Climate Change on the Potential Habitats of Two Medicinally Important *Aconitum* Species in the Hindu Kush Himalaya

**DOI:** 10.1002/ece3.72965

**Published:** 2026-01-21

**Authors:** Uttam Babu Shrestha, Shirish Maharjan, Achyut Tiwari, Yan Luo, Phuentsho, Suresh Kumar Ghimire, Bharat Babu Shrestha

**Affiliations:** ^1^ Global Institute for Interdisciplinary Research (GIIS) Kathmandu Nepal; ^2^ Central Department of Botany, Institute of Science and Technology Tribhuvan University Kathmandu Nepal; ^3^ Southeast Asia Biodiversity Research Institute, Chinese Academy of Sciences & Center for Integrative Conservation, Xishuangbanna Tropical Botanical Garden Menglun, Mengla Yunnan China; ^4^ National Biodiversity Centre, Ministry of Agriculture and Livestock Royal Government of Bhutan Serbithang, Thimphu Bhutan

**Keywords:** *Aconitum naviculare*, *Aconitum spicatum*, allopatric species, habitat suitability, mountain medicinal plants, niche overlaps, species distribution modeling

## Abstract

Climate change is a major driver influencing species survival and distribution, particularly for species endemic to mountainous regions. The Hindu Kush Himalaya (HKH), which is a global biodiversity hotspot and the world's youngest mountain system with a high level of endemism, is particularly vulnerable to climate change impacts. This study investigates how future climate change may affect the potential distribution of two congeneric species, *Aconitum spicatum* and *Aconitum naviculare*, both endemic to the HKH and occupying habitats with contrasting moisture regimes. Using machine learning‐based ecological niche modeling, we assessed projected changes in climatically suitable habitats under moderate (SSP2‐4.5) and high (SSP5‐8.5) emissions scenarios for two future epochs. Our results indicate that *A. spicatum* , which prefers moist environments, is projected to experience a decline in suitable habitats across much of its current range without a shift of projected elevation range, particularly in China, India, and Myanmar, due to warming and altered precipitation patterns. Conversely, *A. naviculare*, which inhabits semi‐arid regions, is expected to exhibit an overall expansion of suitable habitats with a shift of projected elevation range, particularly in China and, to a lesser extent, Nepal, suggesting potential emergence of new ecological niches under future climate conditions. These contrasting responses highlight the species‐specific nature of climate change impacts. Additionally, the overlapped suitable habitat areas of these two species are predicted to decline in future. While future climate change may offer new opportunities for range expansion of the currently range‐restricted *A. naviculare*, it may simultaneously shrink the habitat range of the more widely distributed *A. spicatum* . Suitable habitat overlaps under current and future climate scenarios of congeneric but allopatric species that we report can have ecological and evolutionary implications. These insights are critical for designing adaptive, species‐specific conservation strategies that integrate both climate projections and socioecological pressures, such as overharvesting.

## Graphical abstract

Mountain endemic plants are likely to be affected significantly by climate change. Using species distribution models, we predicted suitable habitat areas of medicinally important and allopatric *Aconitum spicatum* and *A. naviculare* encompassing their entire distribution in the Hindu‐Kush Himalaya and showed that the suitable area will decline without change in elevation range for the species naturally found in wet region ( *A. spicatum* ) but will expand with elevation range shift for the species found in semi‐arid region (*A. naviculare*). These allopatric species also have their suitable areas overlapped. These insights are critical for designing adaptive, species‐specific conservation strategies that integrate both climate projections and socioecological pressures.

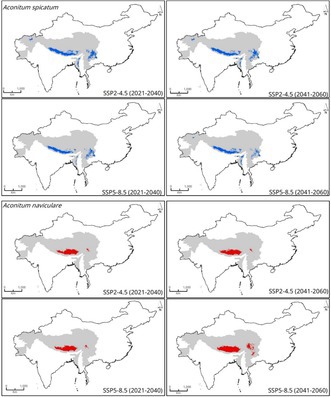

## 1 Introduction

Climate change is one of the direct drivers of biodiversity loss (IPBES 2019), influencing phenology (Richardson et al. 2018), altering species distributions (Parmesan 2006), and increasing extinction risks (Román‐Palacios and Wiens 2020). Mountain regions, which harbor one‐third of the world's terrestrial species diversity (Körner 2004) and preserve signatures of long‐term eco‐evolutionary processes (Rahbek et al. 2019), are particularly vulnerable as climate change reduces the ecological resilience of mountain ecosystems by disrupting their ecosystem functions (Peters et al. 2019). The biodiversity of the Hindu Kush Himalaya (HKH)—a global biodiversity hotspot and the youngest mountain system—is particularly at risk, as the HKH region is experiencing accelerated warming, glacial melt, and altered precipitation patterns with torrential monsoons and extended winter droughts (Immerzeel et al. 2010; Shrestha et al. 2012; Mittal et al. 2014; Mayewski et al. 2020). These climate‐driven changes pose significant threats to the region's rich and unique biodiversity, including several endemic and threatened plant species (Xu et al. 2009; Chettri et al. 2010, 2023; Maikhuri et al. 2018). Given the vast geographic extent of the HKH region, its high level of endemism and extreme microclimatic variability, understanding how climate change affects various aspects of biodiversity—including species distributions, community dynamics, and ecosystem functions—is imperative.

The HKH, also known as the “third pole,” is among the regions most affected by climate change outside polar regions (Williams et al. 2007; Mayewski et al. 2020). Studies in the HKH region have documented various climate‐induced changes, including shifts in tree lines (Schickhoff et al. 2015; Sigdel et al. 2024), endemic species' elevational range shift (Telwala et al. 2013), phenological changes at ecosystem (Shrestha et al. 2012) and species (Hart et al. 2014) levels, alterations in species diversity (Salick et al. 2019), and enhanced growth of vegetation at high elevations (Anderson et al. 2020). Local perceptions based on traditional ecological knowledge also report similar trends, such as phenological changes and poleward shifts of species (Chaudhary and Bawa 2011; Xu et al. 2009). Additionally, studies based on species distribution modeling have predicted both expansion (Shrestha and Bawa 2014) and contractions (Rana et al. 2020; Shrestha, Lamsal, et al. 2022) of suitable habitats, as well as distribution shifts toward higher elevation (Kunwar et al. 2023) under future climate change scenarios. Here, we investigate the potential effects of climate change on two congeneric species that share a common evolutionary history but occupy contrasting ecological niches (moist vs. dry). By comparing habitat suitability under current and projected future climate scenarios for these closely related species, we provide insights into niche differentiation, adaptive strategies, and resilience, which are critical for predicting how small evolutionary differences might influence species survival and distribution in the face of rapid climate change.

Species distribution modeling (SDM) has become an essential tool in ecology and biogeography for predicting the potential distribution of species based on correlations between observed occurrence data and environmental variables (Elith et al. 2006). Over time, SDMs have evolved from simple statistical correlations between species and environmental covariates to more sophisticated frameworks, such as maximum entropy (MaxEnt) (Phillips et al. 2006) and ensemble modeling approaches (Thuiller et al. 2009). The SDM approaches are now widely applied in wildlife research, conservation planning, studies of biological invasions, and assessments of climate change impacts (Vasconcelos et al. 2024). They are increasingly used to predict potential species distributions under future climate change scenarios by training models on current climatic and environmental conditions (Bellard et al. 2013). Despite inherent limitations and uncertainties originating from choices among different modeling algorithms, dispersal strategies, global circulation models (GCMs), and greenhouse gas emission scenarios (Thuiller et al. 2019), SDMs provide a basis for understanding species' responses to climate change and for devising proactive conservation measures and adaptive management strategies (Franklin 2010; Dawson et al. 2011). Given the availability of more than 30 algorithms capable of modeling species distributions (Norberg et al. 2019), selecting the most appropriate models can be challenging because each model has strengths and weaknesses. This challenge is often addressed using an ensemble modeling technique that aggregates predictions of different methods (Thuiller et al. 2009). This approach of combining predictions from multiple algorithms can avoid overfitting and biased estimations (Schmitt et al. 2017), capture complex environmental relationships that might be missed by individual models (Grenouillet et al. 2011), and ultimately perform better than standard single models (Araújo and New 2007).

We model the current and future potential distribution of two congeneric species from the genus *Aconitum*, *A. spicatum* Stapf and *A. naviculare* (Brühl) Stapf, which are endemic to the HKH region. Localized studies have reported both expansion and contraction in the distribution of endemic *Aconitum* species across the Himalaya. For example, species distribution modeling of *A. spicatum* in Nepal predicted a significant increase in climatically suitable areas under future scenarios (Rana et al. 2020). In contrast, the suitable habitats of *A. heterophyllum* in Himachal Pradesh (Tomar et al. 2025) and in Jammu, Kashmir, and Ladakh (Wani et al. 2022) are projected to decline under future climate change. Similarly, *A. heterophyllum* and *A. balfourii* are expected to shift their ranges upward and eastward (Chauhan et al. 2025). However, the impact of climate change to this species throughout its entire distribution range in the HKH region remain unknown. Additionally, no similar studies appear to have focused on *A. naviculare*, suggesting a significant knowledge gap. Both species are widely used in traditional medicine across various cultures in the region (Bhandari et al. 2021; Nyirimigabo et al. 2015; Ghimire et al. 2025). *Aconitum spicatum* is one of 30 medicinal plants (MPs) prioritized by the Government of Nepal for national economic development (DPR 2009) and ranks among the highly traded medicinal plants in Nepal (Rana et al. 2020). *Aconitum naviculare*, on the other hand, is not formally traded, however, is the second most frequently used medicinal plant in *Amchi* traditional medicine of the Himalaya (Ghimire et al. 2021). It is considered as the most effective and frequently used plant species by local communities in Manang, Nepal (Shrestha and Dall'Acqua 2011; Ghimire et al. 2025).

Both species face the risk of over‐exploitation, threatening their long‐term survival. Given that climate change is a major driver of biodiversity change, and the HKH is one of the most vulnerable regions to climate change (Adler et al. 2022), understanding the impacts of future climate change on the distribution of these species is crucial for supporting livelihoods, traditional healthcare, cultural heritage and economic benefit of local communities across their distribution range. In this context, the current research is guided by following research questions: (1) What is the current spatial extent of climatically suitable regions for these two allopatric *Aconitum* species ( *A. spicatum* and *A. naviculare*) in the HKH region? (2) Do these two species occupying contrasting ecological niches (moist vs. dry regions), respond similarly to future climate change? and (3) Are there any areas that are climatically suitable for both species in the HKH region? To address these questions, we employed an ensemble species distribution modeling approach to estimate the current extent of suitable habitats for both species and predict spatiotemporal changes in climatically suitable habitats under moderate (SSP2‐4.5) and high (SSP5‐8.5) emissions scenarios across two future time frames (2021–2040 and 2041–2060).

## 2 Materials and Methods

### 2.1 Study Species

**FIGURE 1:**
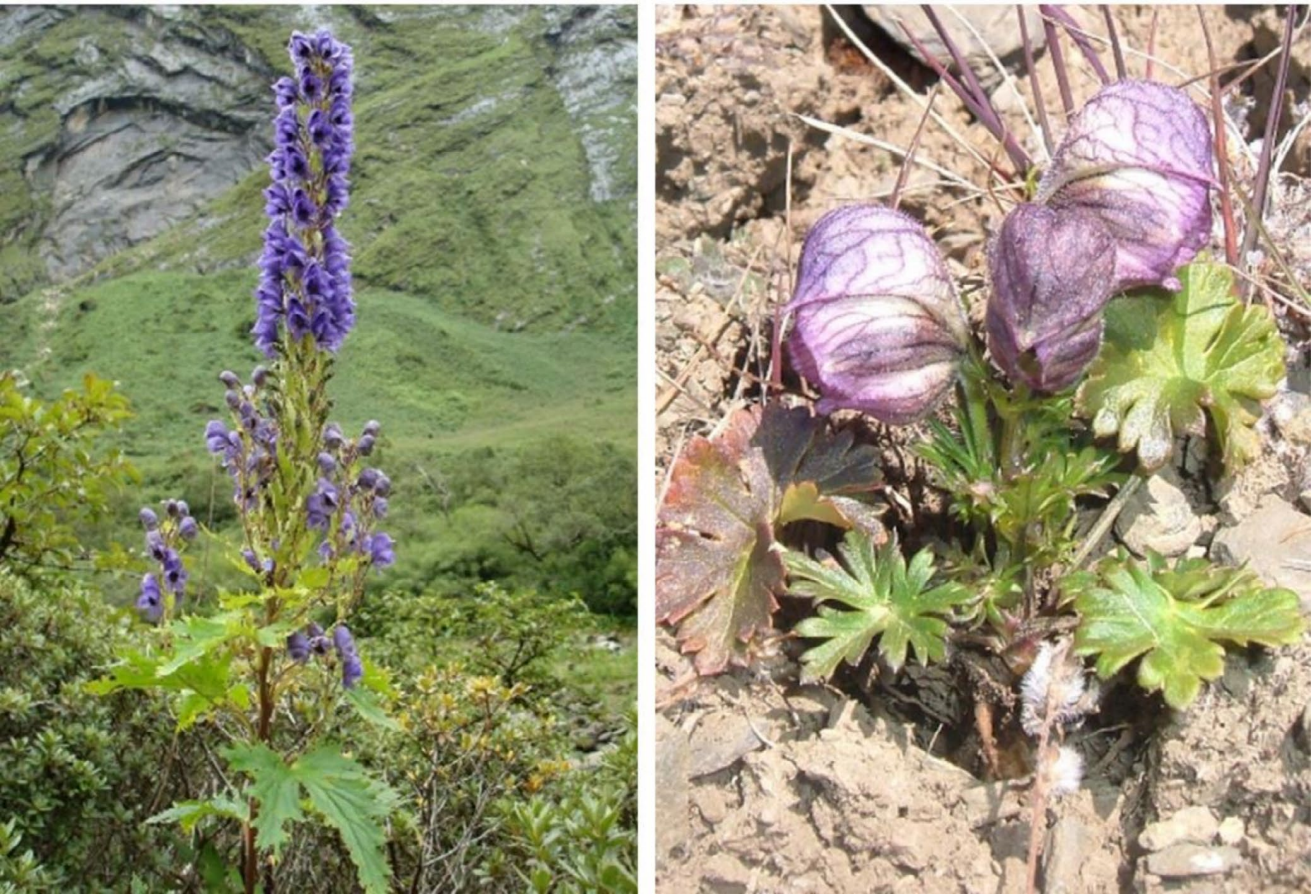
Study species: *Aconitum spicatum* (left) and *Aconitum naviculare* (right).

**FIGURE 2:**
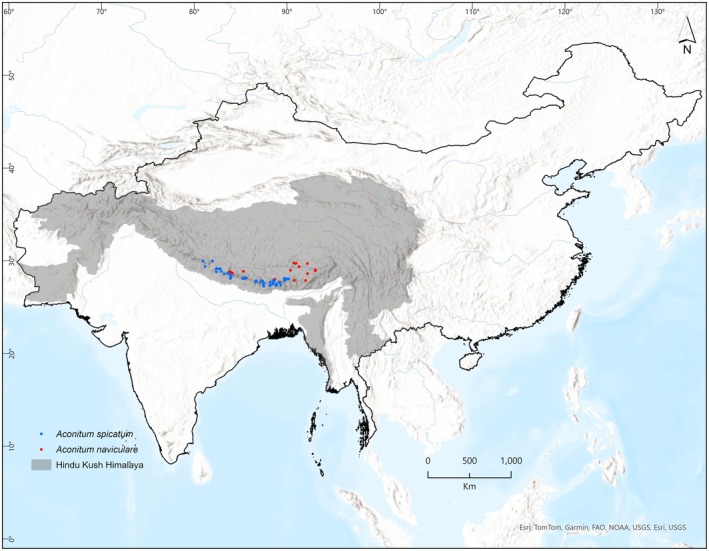
Study area showing occurrence points of *Aconitum spicatum* (blue dots) and *Aconitum naviculare* (red dots).

We selected two endemic mountain medicinal plants of the family Ranunculaceae—*Aconitum spicatum* Stapf and *A. naviculare* (Brühl) Stapf—which are reported from Nepal, Bhutan, India, and China of the Hindu Kush Himalaya (Figures 1 and 2). *A. spicatum* is an extensively traded medicinal plant, whereas *A. naviculare* is highly valued and commonly used by local communities but not in trade. Morphologically, *A. spicatum* is a taller perennial herb (up to 1.5 m) with several flowers in a large terminal inflorescence, whereas *A. naviculare* is a smaller one (up to 45 cm) with few flowers in a terminal inflorescence. *Aconitum spicatum* is typically found in open and damp areas in forests, shrubberies, and grassy slopes at elevations between 3000 and 4800 m asl (Chapagain et al. 2019; Shrestha, Bhandari, and Bhattarai 2022), while *A. naviculare* occurs in alpine meadows and shrublands in semiarid and trans‐Himalayan regions at elevations between 4000 and 4900 m asl (Lama et al. 2001; Cao et al. 2008; Shrestha and Jha 2010). In spite of the broadly overlapping distribution range of these two species, we are not aware of their co‐occurrence in the natural habitats. Traditionally, *A. spicatum* is used for its antipyretic and analgesic properties to treat cough, fever, infections, headaches, and wounds (Lama et al. 2001; Uprety et al. 2010). Likewise, *A. naviculare* is used as a sedative, analgesic, and febrifuge and is employed against fever, headache, jaundice, high blood pressure, cold, gastritis, hepatitis, and nephritis by the local communities (Gao et al. 2004; Cao et al. 2008; Lama et al. 2001; Shrestha and Dall'Acqua 2011; Ghimire et al. 2025).

### 2.2 Species Occurrence Data

This study covers the entire habitat range of *A. spicatum* and *A. naviculare* in the HKH region (Figure 2). We gathered occurrence data from both our field surveys and publicly available records from the Global Biodiversity Information Facility (GBIF). Occurrence records were collected during field visits by the authors: in Nepal by SKG and BBS, in China by Y.L., and in Bhutan by P. We also obtained some occurrence data for India through personal communication with researchers at the University of Kashmir, Jammu and Kashmir, India. Additionally, we downloaded occurrence records for *A. spicatum* (GBIF 2023b) and *A. naviculare* (GBIF 2023a) from the GBIF. Altogether we compiled 88 and 128 occurrence records of *A. spicatum* and *A. naviculare*, respectively. All datasets were merged and thoroughly cleaned by removing erroneous coordinates and duplicate records before analysis.

To mitigate sampling biases resulting from multiple presence locations, we applied spatial filtering techniques using the SDM toolbox 2.3 (Brown 2014). Because our analysis was conducted at a 1 km^2^ resolution, we removed duplicate occurrence records within the same grid cell, retaining one unique record per grid. This filtering process resulted in 64 records for *A. spicatum* and 30 records for *A. naviculare* (Appendix S1), which were subsequently used for species distribution modeling.

### 2.3 Environmental Variables

We collected environmental variables that represent ecological conditions relevant to the species' physiological tolerance, life history traits, and habitat preferences. These included 19 bioclimatic variables, two topographic variables (aspect, elevation), and two soil parameters (soil temperature and soil moisture). Temperature and precipitation are particularly critical for these alpine herbs (Wani et al. 2022; Tomar et al. 2025), as they strongly influence growth, reproduction, and survival at high elevations and heterogeneous landscapes like mountains. We downloaded 19 bioclimatic variables from WorldClim 2 (http://worldclim.org) at a spatial resolution of 30‐arc‐sec (~1 × 1 km) (Fick and Hijmans 2017). These variables were derived from monthly data spanning the period from 1970 to 2000, including key parameters such as minimum, average, and maximum temperatures, along with monthly precipitation data (Hijmans et al. 2005).

We derived soil temperature data from the ERA5 monthly dataset, which provides global data from 1950 to the present (Muñoz‐Sabater et al. 2021). Specifically, we used soil temperature level 1, representing the 0–7 cm depth layer. Soil moisture data were obtained from the TerraClimate dataset, which includes global hydrological and climate data from January 1958 onward (Abatzoglou et al. 2018). To ensure temporal consistency with the WorldClim bioclimatic variables, we used average values taken from the monthly data from the 1970–2000 period. Aspect was calculated from the NASA SRTM (Shuttle Radar Topography Mission) Digital Elevation Model (DEM) (NASA JPL 2013). All spatial analyses were performed in Google Earth Engine, and all environmental layers were resampled to match the original resolution and extent (1 × 1 km) of the bioclimatic variables.

To avoid multicollinearity, which could lead to biased results, we removed environmental variables that displayed high correlations applying Variance Inflation Factor (VIF). We removed environmental variables with VIF values greater than 10 (Dormann et al. 2013). As a result, we retained eight bioclimatic variables for further analysis: mean diurnal range (BIO2), isothermality (BIO3), mean temperature of wettest quarter (BIO8), mean temperature of driest quarter (BIO9), precipitation of the driest month (BIO14), precipitation seasonality (BIO15), precipitation of warmest quarter (BIO18), and precipitation of the coldest quarter (BIO19). These eight bioclimatic variables and an additional one soil (soil moisture) and one topographic (aspect) variable were used as predictors to model the current and future distributions of the two *Aconitum* species.

For future climatic projections, we selected two distinct periods (2021–2040 and 2041–2060) and examined them under two shared socio‐economic pathways (SSP2‐4.5 and SSP5‐8.5). The SSP2‐4.5 represents a middle‐of‐the‐road scenario with no drastic shifts in social, economic, and technological trends from historical patterns. Under this scenario, global warming is projected to reach approximately 2.0°C during the period 2041–2060 and 2.7°C during the period 2081–2100 (IPCC 2021). On the other hand, the SSP5‐8.5 represents the most extreme scenario, which is driven by high greenhouse gas emissions. Under this pathway, significant warming is projected, reaching 2.4°C for 2041–2060 and a staggering 4.4°C for 2081–2100 (IPCC 2021).

Future bioclimatic variables were derived from the Coupled Model Intercomparison Project Phase 6 (CMIP6) outputs provided by the Intergovernmental Panel on Climate Change (Swart et al. 2019). To ensure robustness and capture variability among models, we generated an ensemble of 14 distinct global circulation models (GCMs) by averaging their values.

### 2.4 Species Distribution Modeling

Numerous modeling approaches are available for predicting species distributions in ecological studies, and no single method is universally superior across all situations, although some models tend to perform better under specific conditions (Valavi et al. 2022). In this study, we utilized the BIOMOD platform using the biomod2 package in R, which is a widely used platform for ensemble modeling of species distributions (Thuiller et al. 2009). Ensemble modeling was chosen to avoid averaging out individual model errors, capture diverse patterns, and improve robustness compared to single modeling algorithms (Araújo and New 2007; Grenouillet et al. 2011; Schmitt et al. 2017). Moreover, the ensemble approach enhances confidence in predictions for rare and data‐poor species, such as the one studied here (Ramirez‐Reyes et al. 2021). However, the default settings in BIOMOD may produce suboptimal models with only average predictive performance, whereas properly optimized ensembles can yield significantly higher accuracy (Valavi et al. 2022). We therefore optimized our models. To build the ensemble model, we combined a suite of algorithms that included three regression methods (GAM: Generalized Linear Model, GLM: Generalized Additive Model, MARS: Multivariate Adaptive Regression Splines), three machine learning methods (ANN: Artificial Neural Network, GBM: Gradient Boosting Machine, RF: Random Forest, XGBoost: Extreme Gradient Boosting), and three classification methods (CTA: Classification Tree Analysis, FDA: Flexible Discriminant Analysis, SRE: Surface Range Envelope).

Each model was trained using 70% of the presence points and evaluated on the remaining 30%, across three replicates. We used prevalence correction (0.5) and variable importance was assessed using five permutations per model. We used two evaluation measures: the area under the curve (AUC) of the receiver operating characteristics (ROC) and the true skills statistics (TSS) for model validation and to assess the model's predictive performance. We selected ensemble models with good predictive accuracy (TSS > 0.7 and AUC > 0.8) based on criteria established in previous studies (Thuiller et al. 2009; Gallien et al. 2012; Bellard et al. 2013). Following the model evaluation, we transformed the species' probability of occurrence into a binary raster format using the maximum TSS threshold (Allouche et al. 2006). Based on these outputs, we estimated the spatial extent of climatically suitable habitats for both species under current and future climate conditions, with results disaggregated by country. We also determined regions that are suitable for both species under current and future climate scenarios.

## 3 Results

### 3.1 Current Suitable Habitats

**FIGURE 3:**
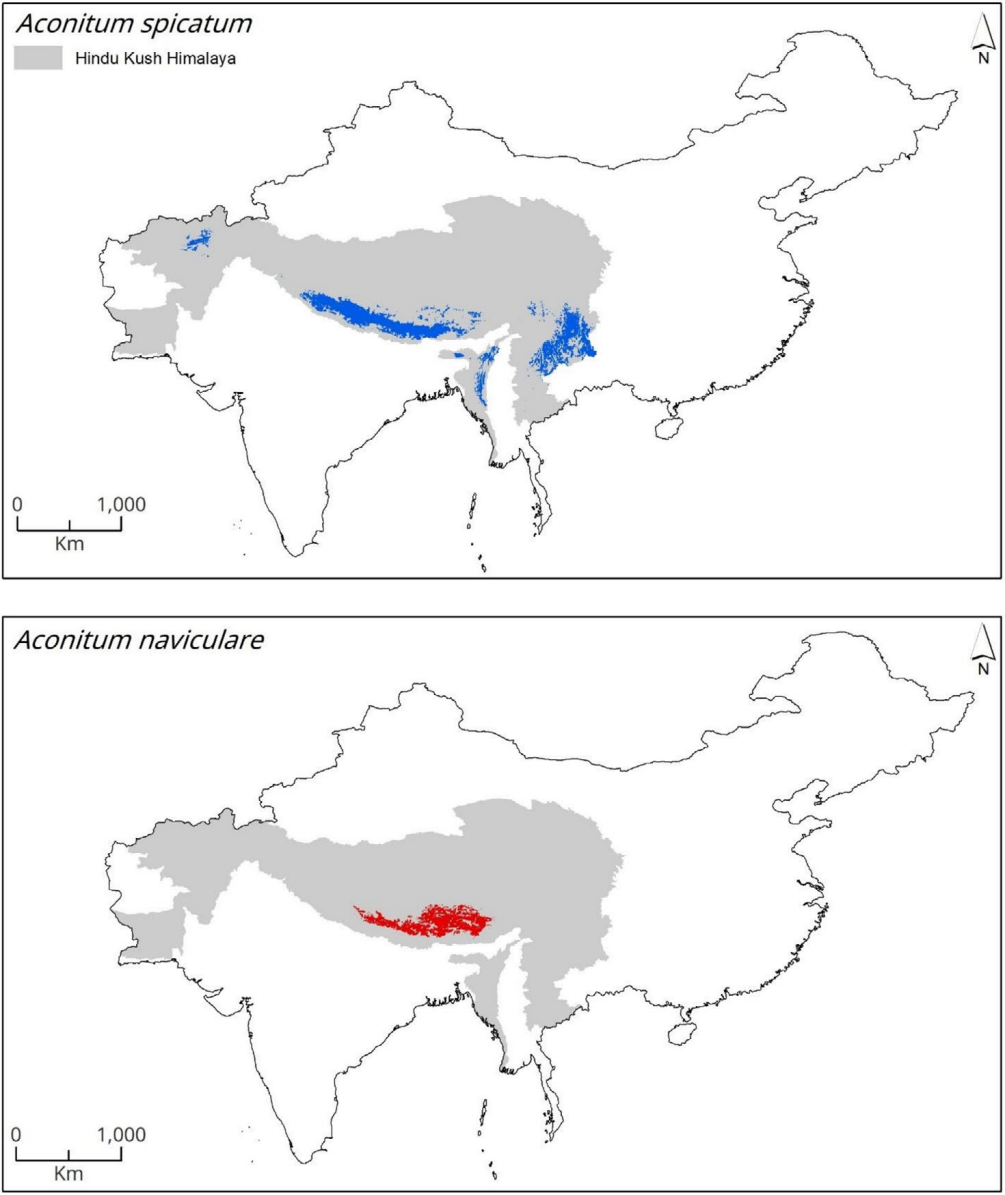
Estimated climatically suitable habitats for *Aconitum spicatum* and *Aconitum naviculare* under current climate.

The ensemble model shows that under current climatic conditions, there are striking differences in the suitable habitats of *A. spicatum* and *A. naviculare*. Approximately 290,323 km^2^ in the HKH region is considered suitable for *A. spicatum* (Figure 3). Similarly, our model predicts that the current suitable area for *A. naviculare* in the HKH region is 111,679 km^2^ (Figure 3). At the country level, China has the largest suitable habitat for *A. spicatum* , accounting for 37% (108,684 km^2^) of the total area, followed by Nepal, which constitutes 33% (96,916 km^2^) of the total area. Pakistan and Afghanistan have minimal suitable areas (Table 1). Likewise, for *A. naviculare*, China currently supports the majority of suitable habitats, covering 80% (89,142 km^2^), followed by Nepal with 16,113 km^2^ (14%). Bhutan and India have a smaller area, while Myanmar and Pakistan have the least suitable habitats as per the prediction of suitable habitat of this species (Table 2).

**TABLE 1:** Climatically suitable areas (km^2^) and percentages for *Aconitum spicatum* under current and future climate conditions.

	Current	SSP2‐4.5		SSP5‐8.5	
		2021–2040	2041–2060	2021–2040	2041–2060
Afghanistan	6922 (2.38%)	3998 (1.85%)	3187 (1.54%)	0 (0%)	747 (0.41%)
Bhutan	17,635 (6.07%)	17,649 (8.19%)	18,523 (8.96%)	13,313 (8.65%)	16,274 (8.98%)
China	108,684 (37.43%)	62,826 (29.17%)	53,612 (25.95%)	32,880 (21.37%)	35,818 (19.76%)
India	52,181 (17.97%)	33,368 (15.49%)	40,641 (19.67%)	24,143 (15.69%)	38,048 (20.99%)
Myanmar	7967 (2.74%)	7665 (3.55%)	4221 (2.04%)	1067 (0.69%)	4354 (2.4%)
Nepal	96,916 (33.38%)	89,804 (41.7%)	86,352 (41.8%)	82,402 (53.57%)	85,950 (47.43%)
Pakistan	18 (0.006%)	10 (0.005%)	0 (0%)	0 (0%)	0 (0%)

**TABLE 2:** Suitable habitats (km^2^) and percentages for *Aconitum naviculare* under current and future climate conditions.

	Current	SSP2‐4.5		SSP5‐8.5	
		2021–2040	2041–2060	2021–2040	2041–2060
Bhutan	3782 (3.38%)	5454 (2.85%)	4905 (2.6%)	5197 (2.58%)	9390 (2.85%)
China	89,142 (79.81%)	161,642 (84.47%)	160,360 (85%)	167,739 (83.43%)	277,037 (84.31%)
India	2632 (2.35%)	4122 (2.15%)	3752 (1.98%)	3496 (1.73%)	6362 (1.93%)
Myanmar	1 (0.001%)	6 (0.003%)	103 (0.05%)	0 (0%)	1861 (0.56%)
Nepal	16,113 (14.42%)	20,126 (10.51%)	19,513 (10.34%)	24,608 (12.24%)	33,850 (10.3%)
Pakistan	9 (0.008%)	0 (0%)	8 (0.004%)	2 (0.001%)	75 (0.02%)

### 3.2 Changes in Suitable Habitats Under Future Climate

**FIGURE 4:**
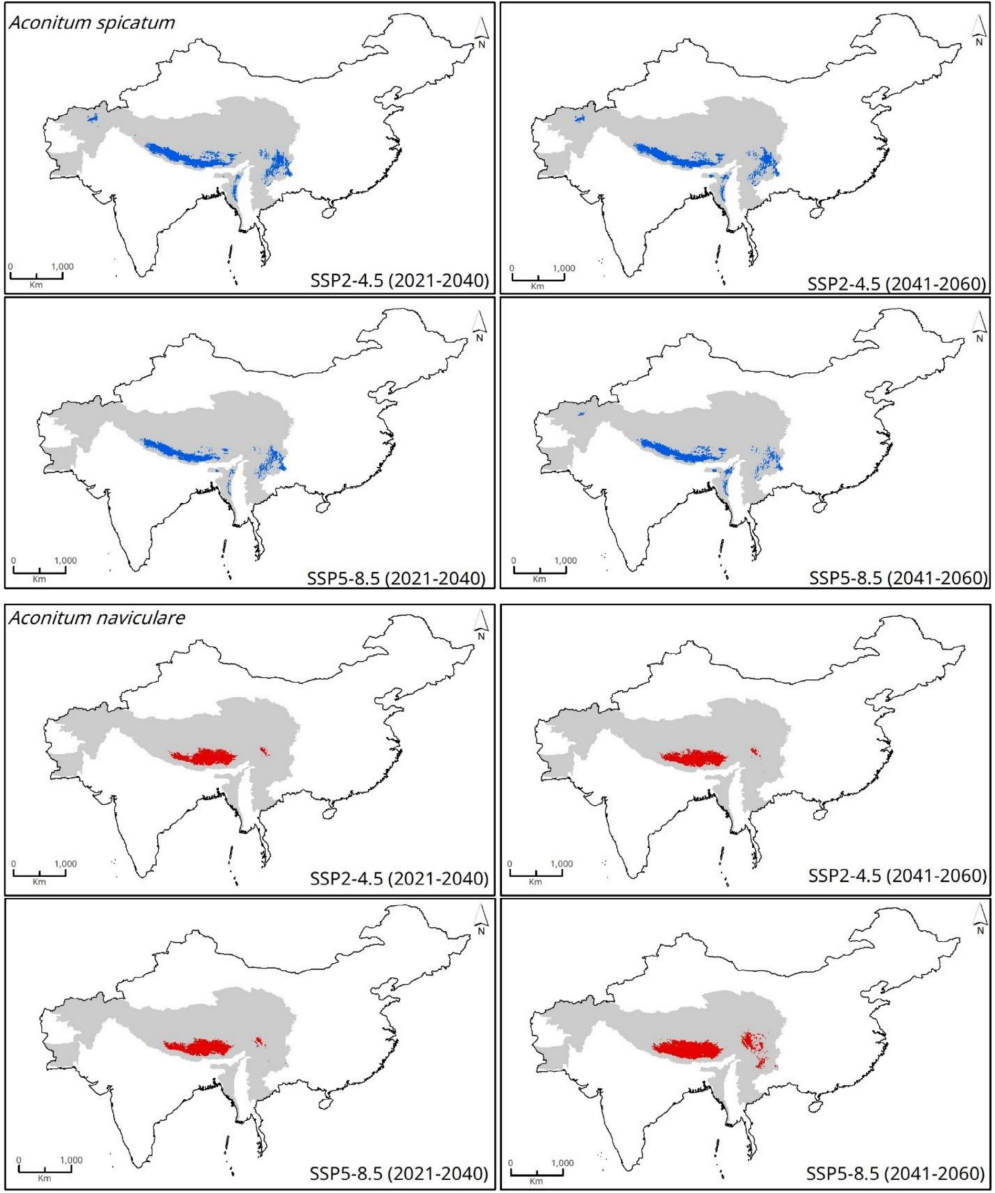
Estimated climatically suitable habitats for *Aconitum spicatum* and *Aconitum naviculare* under two future scenarios (SSP2‐4.5 and SSP5‐8.5) in two different time periods (2021–2040) and (2041–2060).

**FIGURE 5:**
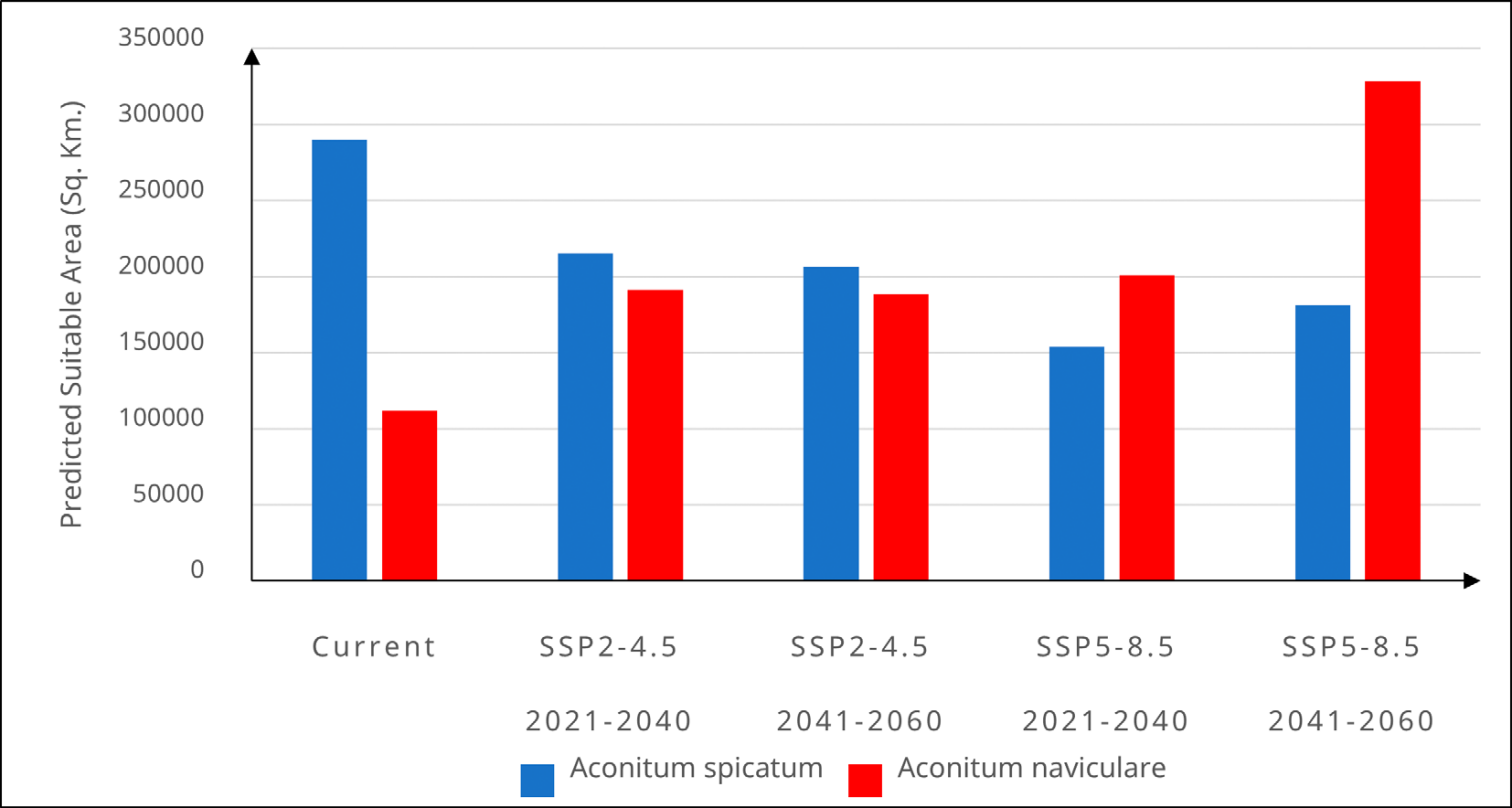
Change in the total suitable habitat areas of *Aconitum spicatum* and *Aconitum naviculare* under current and future climatic conditions.

The future ensemble models show that there will be a decrease in the habitat suitability of *A. spicatum* and an increase in *A. naviculare* (Figures 4, 5). Under the moderate emissions scenario (SSP2‐4.5), the total suitable habitat of *A. spicatum* decreases to about 215,320 km^2^ in 2021–2040 and further to around 206,536 km^2^ in 2041–2060. Under the high emissions scenario (SSP5‐8.5), the reduction of suitable habitats is even more pronounced, with a decline to approximately 15,3805 km^2^ in 2021–2040, followed by a modest recovery to about 181,191 km^2^ in 2041–2060.

At the country level, under the SSP2‐4.5 scenario, suitable habitats of *A. spicatum* are predicted to decline drastically in China from 108,684 km^2^ under current conditions to 62,826 km^2^ by 2021–2040 and further decline to 53,612 km^2^ by 2041–2060. Nepal shows a similar consistent decline, from 96,916 km^2^ to around 89,804 km^2^ and then to 86,352 km^2^ over the same periods. India and Myanmar also exhibit reductions in suitable habitats, while Bhutan shows a minor increase (Table 1). Under the SSP5‐8.5 scenario, the projected decline in suitable habitat for *A. spicatum* is even more pronounced in China, with a decline to 32,880 km^2^ in the near term (2021–2040) and then a rebound to 35,818 km^2^ by the far‐future period (2041–2060). Nepal, Bhutan, India, and Myanmar observe similar trends, declining in 2021–2040 and then increasing in 2041–2060, resulting in a significantly reduced overall suitable area compared to current conditions.

In contrast, *A. naviculare*, which currently occupies a much smaller habitat area than *A. spicatum* , is projected to expand its distribution under future climate scenarios. Under the moderate emission scenario (SSP2‐4.5), the total climatically suitable area is expected to increase to about 191,350 km^2^ by 2021–2040, followed by a slight decline to around 188,641 km^2^ by 2041–2060, though it remains higher than the current habitat area (Figures 4 and 5). Similarly, under the extreme scenario (SSP5‐8.5), suitable habitats are predicted to expand even more dramatically, reaching about 201,042 km^2^ in 2021–2040 and further expanding to approximately 328,575 km^2^ by 2041–2060 (Figure 5).

At the country level, under SSP2‐4.5 scenario, the suitable habitat area for *A. naviculare* is projected to increase considerably in China, reaching 161,642 km^2^ (2021–2040) before slightly contracting to 160,360 km^2^ in 2041–2060. Suitable habitats in Nepal are expected to expand to 20,126 km^2^ in the near term (2021–2040) but then slightly decline to 19,513 km^2^ later in 2041–2060. Bhutan and India show minor fluctuations in habitat suitability across the projected periods, while Myanmar and Pakistan are also predicted to show fluctuations despite having bare minimum suitable habitats under current and future periods.

Under the SSP5‐8.5 scenario, country‐specific trends for *A. naviculare* show considerable variation. In China, the climatically suitable habitat area is projected to increase to 167,739 km^2^ in 2021–2040 and further expand to 277,037 km^2^ by 2041–2060. Suitable habitats in Nepal are also expected to expand significantly, reaching 24,608 km^2^ in 2021–2040 with a more pronounced increase to 22,850 km^2^ in the later period. Bhutan and India show similar trends, though the changes are modest in scale (Table 2).

### 3.3 Change in Elevation Range

**FIGURE 6:**
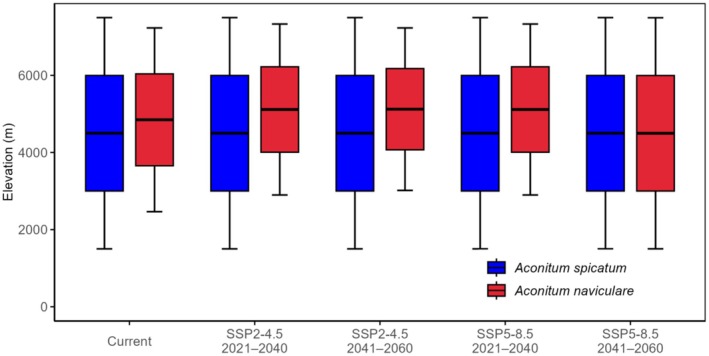
Box plots showing changes in elevation range of suitable habitats of *Aconitum spicatum* and *Aconitum naviculare* under current and future climates.

The projected changes in elevation range of *A. spicatum* and *A. naviculare* under current and future climate scenarios are given in Figure 6. On average, the elevation range of *A. spicatum* is lower than that of *A. naviculare*. Overall, there is an upward shift in average elevation for *A. naviculare* under future climate conditions, but not for *A. spicatum* .

For *A. naviculare*, the current median elevation is 4847 m, and a significant portion of suitable habitats is clustered around 4000–5000 m. This species is projected to shift upward under moderate warming (SSP2‐4.5), and its range could both expand upslope and downslope under a more extreme scenario (SSP5‐8.5), potentially reflecting either wider climatic tolerance, new suitable microclimates, or model uncertainty at range edges. However, *A. spicatum* shows no such significant shift under future climate scenarios.

### 3.4 Overlapped Suitable Areas

**FIGURE 7:**
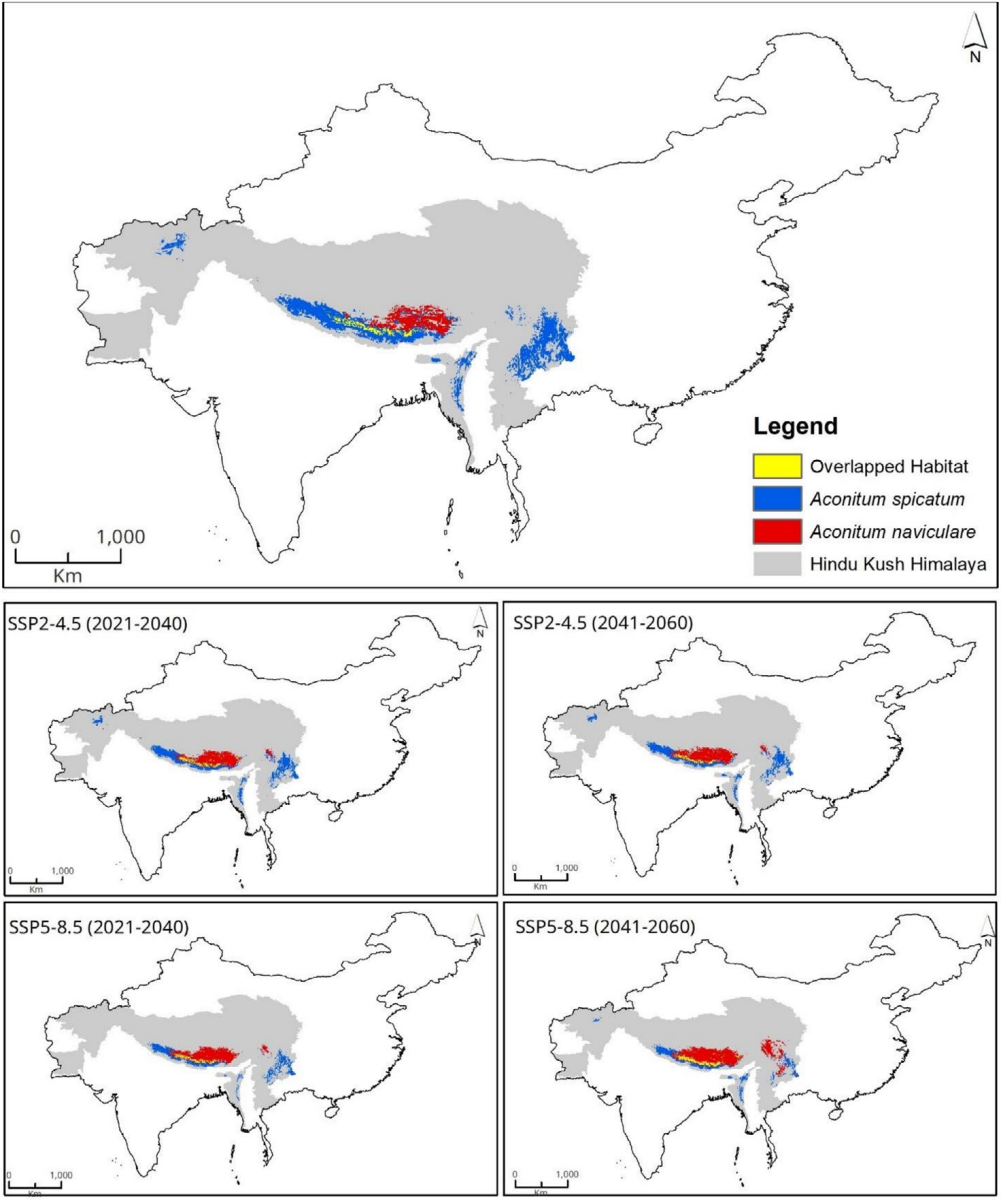
Overlapped habitats between two *Aconitum* species under current and future climates.

Under current climatic conditions, the suitable habitat overlap between the two species covers approximately 31,763 km^2^, which accounts for 11% and 28% of the climatically suitable areas of *A. spicatum* and *A. naviculare*, respectively (Figure 7). The maximum area of overlap was found in Nepal (15,016 km^2^), followed by China (11,793 km^2^), Bhutan (3660 km^2^), and India (1294 km^2^). However, the overlapping area is projected to contract constantly over time, but the extent of contraction differs with the climate scenarios and time. Under the moderate emission scenario (SSP2‐4.5), the projected overlapped habitats decline to about 29,629 km^2^ during 2021–2040, and further decrease to roughly 27,931 km^2^ by 2041–2060 (Figure 7). In contrast, under the high emission scenario (SSP5‐8.5), the estimated overlapping habitat is projected to decline to around 26,163 km^2^ in 2021–2040, but then shows a rebound to approximately 28,324 km^2^ by 2041–2060.

## 4 Discussion

We have analyzed the climatically suitable habitats for two allopatric species of *Aconitum* ( *A. spicatum* and *A. naviculare*) endemic to the HKH region encompassing their entire distribution range for the first time. Our ensemble species distribution models illustrate the complexity of climate change impacts, demonstrating that even closely related congeners can exhibit markedly different trajectories under similar environmental pressures. While *A. spicatum* is projected to experience a significant decline in climatically suitable habitats, without a shift in elevation range, except for a modest rebound under the SSP5‐8.5 scenario in the 2041–2060 period, *A. naviculare* shows potential for expansion as well as elevation range shift under future climate change with only a minor decline projected under SSP2‐4.5 in 2041–2060 relative to the previous period. These non‐linear patterns of gain and loss reflect the ecological complexity and dynamic nature of climate systems. For example, a small increase in temperature may initially lead to habitat loss by surpassing physiological thresholds whereas a more pronounced change can later create new areas of suitability at higher elevations. Additionally, the more dramatic climatic shifts projected for the later time periods may further alter the spatial configuration of suitable habitats. Nevertheless, these findings have significant implications for conservation planning and biodiversity management in the HKH, highlighting the need for species‐specific strategies and long‐term monitoring.

The most noteworthy result of the present study is a contrasting dynamic of the climatically suitable areas of the two species under future climate scenarios. The results clearly show a future decline in climatically suitable regions of *A. spicatum* , which is naturally found in wet regions. In contrast, the climatically suitable region is predicted to increase for congeneric *A. naviculare*, which is naturally found in the semi‐arid regions of the HKH. Contrasting responses to climate change by phylogenetically distant but co‐occurring high mountain plants have also been reported previously (Mainali et al. 2020; Sigdel et al. 2024). In particular, species preferring dry microhabitat, such as *Abies spectabilis*, benefited more from warming than species that prefer moist microhabitat, such as *Betula utilis* (Sigdel et al. 2024). A species distribution modeling of three species of *Bergenia* in the Himalaya‐Hengduan Mountains region revealed that the climatically suitable area of the species of relatively dry Western Himalaya (*Bergenia stracheyi*) is likely to expand, while the species of relatively moist Eastern Himalaya‐Hengduan ( *B. purpurascens* ) is likely to witness a net loss of the suitable area in the future (Qiu et al. 2024). Additionally, a recent study by Chondol et al. (2025) also highlights the major roles played by habitat conditions in determining interspecific plant growth variations, with plants of dry microhabitats showing higher radial growth rates than the plants found in wet microhabitats of the Western Himalaya. Results of the present and several previous studies (as discussed above) suggest a higher vulnerability of mountain plants inhabiting moist microhabitats to climate change than those inhabiting dry microhabitats. This kind of habitat‐associated and species‐specific responses to climate change need to be accounted for during conservation planning, as pre‐monsoon drought episodes are likely to increase in the future due to the increased temperature and reduced precipitation in the high mountain regions of the Himalaya (Pepin et al. 2022).

Previous studies have shown that species respond to climate change by shifting their distributions, particularly toward higher elevations (Lenoir et al. 2008; Parmesan 2006; Qiu et al. 2024; Chauhan et al. 2025). However, the degree of this response varies, with species residing in areas experiencing greater warming exhibiting substantially greater range shifts (Chen et al. 2011). The warming rate in the Himalayan region is higher than the global average (Kohler and Maselli 2009; Shrestha et al. 2012; IPCC 2013), so it is no surprise that suitable habitats for *A. naviculare* species are shifting upwards. A similar modeling study has also shown the upward elevational shift of medicinal plants in Himalaya (Kunwar et al. 2021), including congeneric species of *A. heterophyllum* (Chauhan et al. 2025). Nevertheless, the magnitude of these elevation shifts differs between species under future climate scenarios, a pattern also reported for other Himalayan species such as *Bergenia* spp. (Qiu et al. 2024). As rising temperatures at lower elevation regions make those regions unsuitable while simultaneously opening previously uninhabitable areas in higher elevations, species are forced to seek cooler conditions (Chen et al. 2011). Satellite data showed a significant greening trend in the Everest region, particularly in the subnival zones (4150–6000 m), where vegetation cover has expanded by 45% from 1993 to 2018, with the highest increase observed in autumn (Anderson et al. 2020). The ability to shift ranges toward higher elevations in response to the declining suitability of their historical habitats due to climate change has also been reported in other studies from the Indian Himalaya (Telwala et al. 2013) and Mt. Gongga of Sichuan, China (Zu et al. 2021). Modeling‐based studies in Nepal (Shrestha, et al. 2022), in Himalaya biodiversity hotspots (Liu et al. 2025), and in the Himalaya‐Hengduan Mountains (He et al. 2019) also showed similar results. However, while an upward shift toward higher elevation is an adaptation response, this does not guarantee species survival; local population declines may still occur due to other factors such as increased competition and limited resource availability (Ohler et al. 2020), aspects that are not considered in our models. Some medicinal plants in central Himalaya showed a decline in natural populations despite having substantial suitable habitats as indicated by modeling results (Kunwar et al. 2020). In a similar modeling study by Ranjitkar et al. (2016), the distribution of medicinal plants of the temperate region of Nepal Himalaya was found to be primarily influenced by elevation, temperature, and precipitation, and a rapidly changing climate was described as the major threat to medicinal plants.

Climatically suitable areas of two species overlap substantially with slightly more than a quarter (28%) of the suitable areas of *A. naviculare* being predicted to be also suitable for *A. spicatum* . Such overlap of suitable habitats despite no known co‐occurrence (i.e., allopatric) of these two congeneric species with adjacent native range can be attributed to the fact that the potential niches are always larger than realized niches (Soberón and Arroyo‐Peña 2017). While the present results indicate a decline in the spatial extent of the overlapped suitable areas in future, other studies have reported climate‐induced niche overlaps of allopatric and congeneric species in the future (Krosby et al. 2015). Whether climate induced or otherwise, suitable habitat overlaps of congeneric allopatric species can have direct ecological (e.g., competition) and evolutionary (e.g., hybridization) implications. For example, pollination studies of these two species in different regions of Nepal Himalaya revealed that their primary pollinators are two different bumble bee species: *Bombus miniatus* Bingham, 1897 of *A. spicatum* and *B. rufofasciatus* Smith, 1852 of *A. naviculare* (Masrangi et al. 2026). Given that bumble bees are generalist pollinators of *Aconitum* species, the same species of bumble bee may pollinate multiple *Aconitum* species and *vice‐versa*, if they share habitats (Utelli and Roy 2000), with possibilities of hybridization (Campbell et al. 2002).

The two *Aconitum* species are subjected to serious harvesting pressure due to increasing trade ( *A. spicatum* ) (Smith‐Hall et al. 2025) and local uses (*A. naviculare*) (Ghimire et al. 2025). Excessive and unsustainable harvesting of medicinal plants in the Himalaya has already negatively impacted their populations (Ghimire et al. 2008; Shrestha and Bawa 2013; Smith‐Hall et al. 2025). Consequently, the combination of detrimental harvesting practices and the projected change in climatically suitable areas will have a cascading effect. Therefore, future conservation strategies in the HKH region must account for the complex interactions between climate change and anthropogenic pressures, such as overharvesting. Our results contribute valuable insights that can inform more effective, species‐specific conservation strategies in this area.

This study has some limitations that should be considered when interpreting our results. First, our species distribution models (SDMs) do not incorporate biotic factors—such as biotic interactions or potential evolutionary adaptation—that could significantly influence species distributions in natural settings. Additionally, because our projections are based solely on SDM modeling, validation of the model predictions with field observations remains a critical next step. Despite these limitations, our results provide multiple lines of evidence for species‐specific responses of congeneric plants to climate change. We emphasize that these findings have significant implications for conservation planning and biodiversity management in the HKH, highlighting the need for habitat‐ and species‐specific strategies and long‐term monitoring.

## 5 Conclusion

This study demonstrated the differences in potential distributions of two congeneric species endemic to the Hindu Kush Himalaya, *A. spicatum* and *A. naviculare*, which are naturally distributed in moist and dry habitats, respectively. The machine learning‐based ecological niche modeling showed that even closely related species can exhibit divergent responses to climate change. *A. spicatum* is projected to experience an overall decline in suitable habitats without elevation range shift under future climate scenarios, with the exception of a rebound under the SSP5‐8.5 scenario in the 2041–2060 period. In contrast, *A. naviculare* is expected to exhibit the expansion of suitable habitats with elevation range shift across most scenarios, with only a minor decline projected under SSP2‐4.5 in 2041–2060, relative to its previous period. The warmer and drier climate in future might create new suitable habitats for the currently range‐restricted *A. naviculare* growing in the dry region, while reducing the suitable habitats for the more widely distributed *A. spicatum* of moist region. Suitable habitat overlaps of congeneric but allopatric species under current and future climate scenarios can have ecological and evolutionary implications. These insights are critical for designing adaptive, species‐specific conservation strategies that integrate both climate projections and socioecological pressures such as overharvesting. Given the limitations of modeling approaches, future efforts should prioritize field validation and incorporate ecological interactions, microhabitat condition to refine conservation actions in this vulnerable mountain ecosystem.

## Author Contributions

**Uttam Babu Shrestha:** conceptualization (equal), data curation (equal), formal analysis (equal), funding acquisition (equal), methodology (lead), validation (equal), visualization (equal), writing – original draft (lead), writing – review and editing (equal). **Shirish Maharjan:** data curation (equal), formal analysis (lead), software (equal), visualization (lead), writing – original draft (equal). **Achyut Tiwari:** conceptualization (equal), data curation (equal), project administration (equal), writing – review and editing (equal). **Yan Luo:** data curation (equal), writing – review and editing (equal). **Phuentsho:** data curation (equal), writing – review and editing (equal). **Suresh Kumar Ghimire:** data curation (equal), writing – review and editing (equal). **Bharat Babu Shrestha:** conceptualization (lead), data curation (equal), funding acquisition (lead), methodology (equal), project administration (lead), supervision (lead), validation (equal), writing – review and editing (lead).

## Funding

B.B.S., A.T., and U.B.S. received funding from the University Grants Commission of Nepal under the Collaborative Research Grant Program (Grant No. CRG‐77/78‐S&T‐1).

## Conflicts of Interest

The authors declare no conflicts of interest.

## Supporting information


**Appendix S1:** ece372965‐sup‐0001‐Appendix1.docx.

## Data Availability

All data used in this research has been presented in Appendix S1 of this manuscript.
