# Supplementary material for: Divergent Effects of Climate Change on the Potential Habitats of Two Medicinally Important Aconitum Species in the Hindu Kush Himalaya
Source: Ecol Evol. 2026 Jan 21;16(1):e72965. doi: 10.1002/ece3.72965 (PMC12823164; doi:10.1002/ece3.72965)
Supplement: Supplementary file 1 — Appendix S1: ece372965‐sup‐0001‐Appendix1.docx. [file ECE3-16-e72965-s001.docx]

# **Appendix**

**Appendix 1**. Final list of occurrence records used for the species distribution modelling. Occurrence records retrieved from the Global Biodiversity Information Facility (GBIF 2023a, b) were combined with those contributed by coauthors and other individual which were subjected to spatial filtering removing all but single occurrence location per 1 km^2^ grid to get the final list of the occurrence records for use in the modelling.

| **SN** | **Latitude (˚N)** | **Longitude (˚E)** |
| --- | --- | --- |
| *Aconitum spicatum* | | |
| 1 | 27.89527 | 88.51112 |
| 2 | 27.33028 | 88.7788 |
| 3 | 27.36111 | 88.14634 |
| 4 | 27.71672 | 88.55392 |
| 5 | 27.3423 | 88.82497 |
| 6 | 27.3672 | 88.22034 |
| 7 | 27.8271 | 88.63091 |
| 8 | 27.51118 | 88.4997 |
| 9 | 27.70764 | 89.28841 |
| 10 | 27.66076 | 89.23695 |
| 11 | 28.11667 | 89.73333 |
| 12 | 28.03333 | 89.78333 |
| 13 | 28.06667 | 89.88333 |
| 14 | 28.04451 | 89.94786 |
| 15 | 28.06667 | 90.18333 |
| 16 | 27.9098 | 89.7302 |
| 17 | 28.0542 | 89.71662 |
| 18 | 27.98221 | 89.73796 |
| 19 | 27.36722 | 89.3484 |
| 20 | 29.98256 | 80.94732 |
| 21 | 29.95858 | 80.96383 |
| 22 | 29.95505 | 80.931 |
| 23 | 29.98063 | 81.97413 |
| 24 | 29.99027 | 81.9827 |
| 25 | 29.12417 | 82.66905 |
| 26 | 29.13983 | 82.82 |
| 27 | 28.49721 | 83.89771 |
| 28 | 28.10112 | 85.3682 |
| 29 | 28.06 | 85.45265 |
| 30 | 28.20483 | 85.56297 |
| 31 | 27.80444 | 86.71417 |
| 32 | 27.82167 | 86.61778 |
| 33 | 27.82472 | 86.72639 |
| 34 | 27.49214 | 88.91574 |
| 35 | 27.52354 | 88.90726 |
| 36 | 27.48649 | 88.82491 |
| 37 | 27.71341 | 88.17906 |
| 38 | 28.52213 | 83.88123 |
| 39 | 28.52838 | 83.89542 |
| 40 | 28.52465 | 83.90782 |
| 41 | 28.50701 | 83.90249 |
| 42 | 28.49096 | 83.89252 |
| 43 | 28.53037 | 83.87641 |
| 44 | 27.82083 | 87.375 |
| 45 | 27.58333 | 87.5 |
| 46 | 28.09806 | 85.19666 |
| 47 | 27.4 | 87.43333 |
| 48 | 28.07389 | 85.42973 |
| 49 | 29.17556 | 82.41306 |
| 50 | 27.71111 | 86.59917 |
| 51 | 27.73333 | 87.18333 |
| 52 | 27.82806 | 87.33056 |
| 53 | 29.145 | 82.36389 |
| 54 | 28.25194 | 83.96917 |
| 55 | 28.30917 | 83.90722 |
| 56 | 28.6 | 83.21667 |
| 57 | 28.655 | 83.615 |
| 58 | 29.38667 | 81.16889 |
| 59 | 28.85083 | 82.94667 |
| 60 | 27.48 | 88.9 |
| 61 | 27.25 | 87.5 |
| 62 | 28.05 | 90.08333 |
| 63 | 28.8 | 82.4 |
| 64 | 28.1 | 83.9 |
| *Aconitum naviculare* | | |
| 1 | 28.66712 | 84.1126 |
| 2 | 28.7458 | 83.98139 |
| 3 | 28.8166 | 83.85 |
| 4 | 28.1706 | 85.61384 |
| 5 | 28.85 | 85.29 |
| 6 | 28.85825 | 85.30206 |
| 7 | 28.1706 | 85.61384 |
| 8 | 28.85825 | 85.30206 |
| 9 | 28.85825 | 85.30206 |
| 10 | 27.89527 | 88.51112 |
| 11 | 28.1706 | 85.61384 |
| 12 | 28.85825 | 85.30206 |
| 13 | 27.89527 | 88.51112 |
| 14 | 28.02703 | 88.69335 |
| 15 | 28.02703 | 88.69335 |
| 16 | 27.72401 | 89.1611 |
| 17 | 28.02703 | 88.69335 |
| 18 | 28.98 | 90.38 |
| 19 | 28.08333 | 89.86667 |
| 20 | 28.98 | 90.38 |
| 21 | 29.78 | 90.73 |
| 22 | 27.9 | 90.78 |
| 23 | 29.37639 | 91.31216 |
| 24 | 29.73 | 90.98 |
| 25 | 27.8856 | 92.01426 |
| 26 | 28.63194 | 92.23194 |
| 27 | 29.70674 | 92.20516 |
| 28 | 27.8856 | 92.01426 |
| 29 | 28.93783 | 93.06325 |
| 30 | 29.04 | 93.07 |
